# Inferior vena cava diameter is associated with prognosis in patients with chronic heart failure independent of tricuspid regurgitation velocity

**DOI:** 10.1007/s00392-023-02178-4

**Published:** 2023-03-10

**Authors:** Antonio Iaconelli, Joe Cuthbert, Syed Kazmi, Pasquale Maffia, Andrew L. Clark, John G. F. Cleland, Pierpaolo Pellicori

**Affiliations:** 1 School of Cardiovascular & Metabolic Health, College of Medical, Veterinary and Life Sciences, University of Glasgow, Glasgow, UK; 2 Fondazione Policlinico Universitario Agostino Gemelli IRCCS, Rome, Italy; 3 Department of Cardiorespiratory Medicine, Centre for Clinical Sciences, Hull York Medical School, University of Hull, Kingston-Upon-Hull, East Riding of Yorkshire, HU6 7RX UK; 4 Department of Cardiology, Castle Hill Hospital, Hull University Teaching Hospitals Trust, Castle Road, Cottingham, Kingston-Upon-Hull, East Riding of Yorkshire, HU6 5JQ UK; 5 School of Infection & Immunity, College of Medical, Veterinary and Life Sciences, University of Glasgow, Glasgow, UK; 6 Department of Pharmacy, School of Medicine and Surgery, University of Naples Federico II, Naples, Italy

**Keywords:** Congestion, Heart failure, Inferior vena cava, Tricuspid regurgitation velocity, Pulmonary hypertension

## Abstract

**Aims:**

A high, Doppler-derived, tricuspid regurgitation velocity (TRV) indicates pulmonary hypertension, which may contribute to right ventricular dysfunction and worsening tricuspid regurgitation leading to systemic venous congestion, reflected by an increase in inferior vena cava (IVC) diameter. We hypothesized that venous congestion rather than pulmonary hypertension would be more strongly associated with prognosis.

**Methods and results:**

895 patients with chronic heart failure (CHF) (median (25th and 75th centile) age 75 (67–81) years, 69% men, LVEF 44 (34–55)% and NT-proBNP 1133 (423–2465) pg/ml) were enrolled. Compared to patients with normal IVC (< 21 mm) and TRV (≤ 2.8 m/s; *n* = 504, 56%), those with high TRV but normal IVC (*n* = 85, 9%) were older, more likely to be women and to have LVEF ≥ 50%, whilst those with dilated IVC but normal TRV (*n* = 142, 16%) had more signs of congestion and higher NT-proBNP. Patients (*n* = 164, 19%) with both dilated IVC and high TRV had the most signs of congestion and the highest NT-proBNP.

During follow-up of 860 (435–1121) days, 239 patients died. Compared to those with both normal IVC and TRV (reference), patients with high TRV but normal IVC did not have a significantly increased mortality (HR: 1.41; CI: 0.87–2.29; *P* = 0.16). Risk was higher for patients with a dilated IVC but normal TRV (HR: 2.51; CI: 1.80–3.51; *P* < 0.001) or both a dilated IVC and elevated TRV (HR: 3.27; CI: 2.40–4.46; *P* < 0.001).

**Conclusion:**

Amongst ambulatory patients with CHF, a dilated IVC is more closely associated with an adverse prognosis than an elevated TRV.

**Graphical Abstract:**

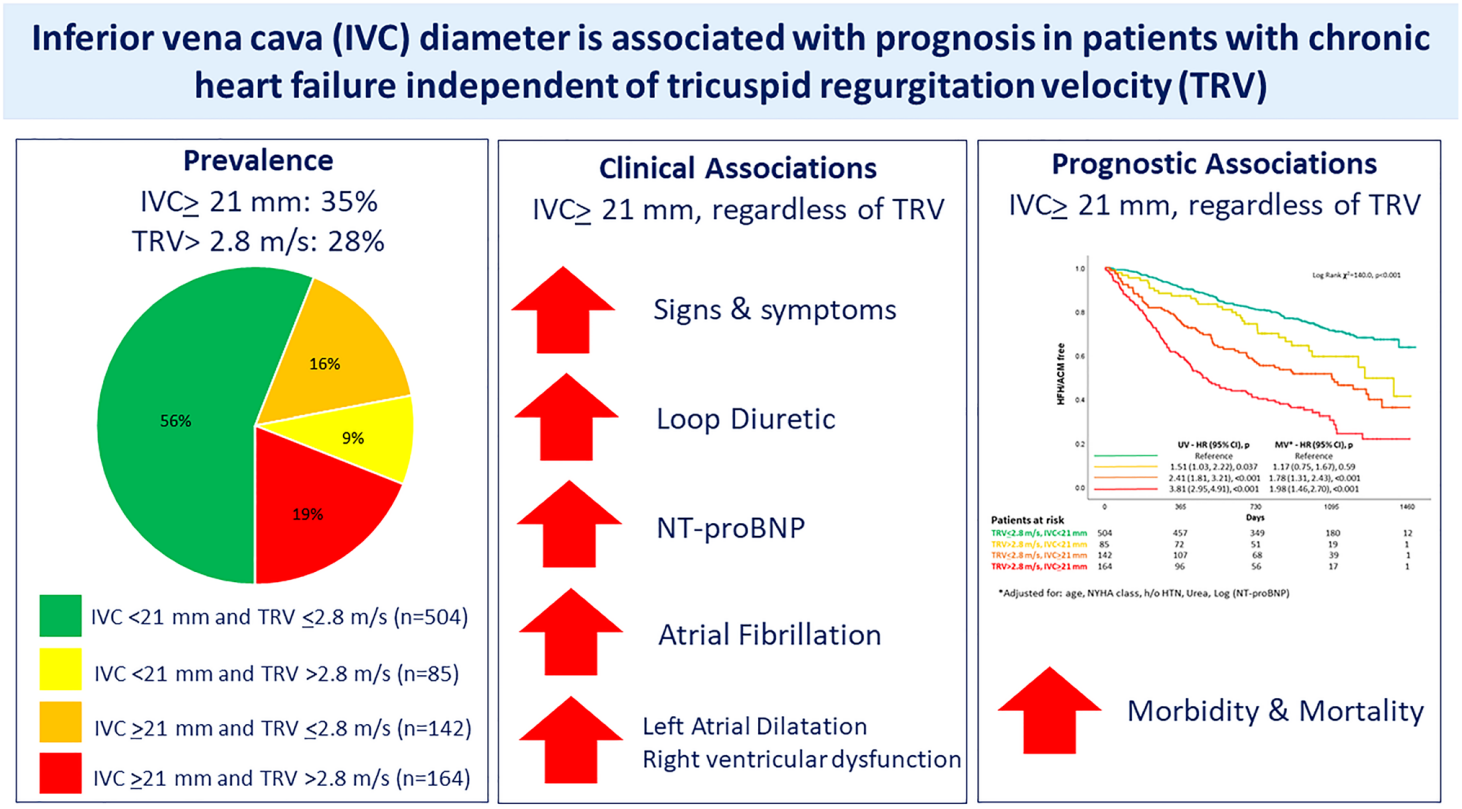

**Supplementary Information:**

The online version contains supplementary material available at 10.1007/s00392-023-02178-4.

## Introduction

Heart failure (HF) is a syndrome caused by cardiac dysfunction and leading to water and salt retention, expanding plasma volume (haemodynamic or circulatory congestion) and increasing water content in many organs (tissue congestion). Most of the increase in plasma volume is accommodated in the venous circulation [1, 2]. Clinical identification and quantification of congestion is difficult, as symptoms and signs are non-specific and usually appear late in the course of disease [3]. Non-invasive imaging techniques can provide objective measurements of both circulatory and tissue congestion that enable early detection and quantification of congestion for patients with, or at risk of, HF [4, 5].

As central venous pressure increases so does the pressure in the pulmonary circulation [6]. Pulmonary hypertension (PHT) is common in patients with HF, especially when congestion is poorly controlled; both PHT and congestion are associated with more severe symptoms and an adverse prognosis [7]. Invasive monitoring of pulmonary artery pressure (PAP) allows for early recognition and treatment of congestion reducing the likelihood of heart failure hospitalisation [8]; however, this technology is currently expensive and the evidence that it is superior to other management strategies is not robust.

Systolic pulmonary artery pressure (sPAP) can be estimated from the tricuspid regurgitation velocity (TRV) on transthoracic echocardiography by adding an approximation of right atrial pressure, but there are difficulties in its interpretation. TRV is a function of the pressure gradient between right ventricle and right atrium in systole, not a direct measure of pulmonary artery pressure, which might be underestimated in the presence of right ventricular dysfunction or severe tricuspid regurgitation. Right atrial pressure can be estimated using other echocardiographic variables [9], but due to the uncertainties in estimating right atrial pressure, recent ESC guidelines on pulmonary hypertension recommend using “*the peak TRV (and not the estimated sPAP) as the key variable for assigning the echocardiographic probability of pulmonary hypertension*” [10].

There is a modest positive correlation between ultrasound markers of venous congestion, such as the inferior vena cava (IVC) diameter, and TRV in patients with HF, but TRV can be normal even when the IVC is markedly dilated [11]. An important function of the venous system is to accommodate a large volume of blood, acting as a reservoir. Due to their high compliance, veins can dilate substantially and buffer an increase in intravascular volume and pressure. An increase in TRV might thus only occur when intravascular congestion is severe, as recent animal experiments suggest [12]. A dilated IVC might identify patients with clinically important venous congestion, even when TRV is normal. Accordingly, we investigated the relationship between IVC diameter and TRV assessed by echocardiography and their associations with prognosis in ambulatory patients with CHF.

## Methods

### Patient population

We enrolled ambulatory patients attending a specialist community heart failure clinic for the initial diagnosis or follow-up in Kingston upon Hull, United Kingdom, between November 2008 and December 2011. HF was defined as the presence of symptoms and signs of the disease and objective evidence of cardiac dysfunction, either left ventricular ejection fraction (LVEF) < 50% or high amino-terminal pro-B-type natriuretic peptide (NT-proBNP) ≥ 125 pg/ml [13].

The study conformed to the principles outlined in the Declaration of Helsinki and was approved by relevant ethical bodies; all participants provided their written informed consent.

Patients provided a detailed clinical history; blood tests, electrocardiograms and echocardiograms were obtained on the same day of the visit. We used the Modification of Diet in Renal Disease (MDRD) equation for the estimation of glomerular filtration rate from plasma creatinine. The minimum follow-up period was 12 months for the last patient enrolled. The outcomes of interest were all-cause mortality and a composite event of admission for worsening HF or all-cause mortality. Data regarding deaths and hospitalisations were collected from the electronic systems of the hospital, supplemented by information from discharge letters, patients and their general practitioner. Hospitalisation for HF was defined as an urgent/emergency admission for worsening of signs/symptoms requiring intensification of treatment with loop diuretics.

### Echocardiographic measurements

Echocardiography was performed by experienced operators with a Vivid Five or Seven (GE Healthcare, Little Chalfont, Buckinghamshire, United Kingdom) system and retrospectively reviewed by a single operator (P.P.) blinded to other patient details. Peak TRV was assessed from the four chamber apical view by Doppler echocardiography and a cut off of 2.8 m/sec was used to discriminate between normal (≤ 2.8 m/s) and high (> 2.8 m/s) values [10]. When TRV was not measurable, the patient was considered to have normal TRV [10]. With the patient supine and from the subcostal view, the maximum IVC diameter during the respiratory cycle was measured approximately 2 cm before it merged with the right atrium. IVC was considered dilated if diameter was ≥ 21 mm [14]. In a subset of patients, (*n* = 680), global longitudinal strain (GLS) was measured using an 18-segment model of the LV, as previously described [15].

For the current analysis, we defined four subgroups of patients with HF: (I) patients with both normal TRV and IVC diameter; (II) patients with elevated TRV but normal IVC diameter; (III) patients with normal TRV and dilated IVC diameter; (IV) patients with both elevated TRV and dilated IVC.

### Statistical analysis

Categorical data are presented as number and proportion (%), continuous data are presented as median (25th and 75th centile). Baseline characteristics between subgroups were compared using chi-squared test for categorical variables. Normally distributed continuous data were compared using one-way analysis of variance (ANOVA) and non-normally distributed continuous data were compared using Kruskal–Wallis test. Kaplan–Meier curves with the log-rank statistic were used to illustrate outcome. We used Cox regression models to investigate the relationship between echocardiographic variables and prognosis, corrected for variables chosen a priori (age, NYHA class, history of hypertension, urea, Log (NT-proBNP)), as these have previously been found to be strongly associated with outcome in the present dataset [11]. All analyses were performed with SPSS (version 26) and a 2-sided p value < 0.05 was considered statistically significant.

## Results

### Demographic and clinical characteristics of the study population

Of the 929 patients enrolled, 34 (4%) were excluded because an IVC diameter could not be measured; of the remainder, an adequate Doppler signal to measure TRV was obtained in 874 patients (98%). Patient characteristics are shown in Table 1. Compared to patients with both a normal IVC and TRV (*N* = 504, 56%), those with high TRV but normal IVC (*N* = 85, 9%) were older and were more likely to be women with a similar prevalence of HF signs and symptoms; those with a dilated IVC but normal TRV (N = 142, 16%) had more signs of congestion and a higher NT-proBNP. Patients with both dilated IVC and high TRV (*N* = 164; 19%), were the oldest, the most congested and had the highest NT-proBNP.

**Table 1:** Baseline demographic characteristics of patients with heart failure (HF) according to tricuspid regurgitation velocity (TRV) and inferior vena cava (IVC) diameter

Variable	TRV ≤ 2.8 m/s IVC < 21 mm (*n* = 504)	TRV > 2.8 m/s IVC < 21 mm (*n* = 85)	TRV ≤ 2.8 m/s IVC ≥ 21 mm (*n* = 142)	TRV > 2.8 m/s IVC ≥ 21 mm (*n* = 164)	*P*-value
Demographics					
Age (years)	73 (64–79)	78 (73–85)	75 (66–81)	79 (74–84)	< 0.001
Men, *n* (%)	347 (69)	47 (55)	109 (77)	110 (67)	0.009
Diabetes, *n* (%)	158 (31)	20 (23)	40 (28)	45 (27)	0.43
BMI (Kg/m^2^)	29.4 (25.8–33.5)	26.4 (23.3–30.1)	27.8 (24.3–31.6)	26.9 (24.1–30.5)	< 0.001
Smoking, *n* (%)	97 (19)	7 (8)	19 (13)	18 (12)	0.009
Hypertension, *n* (%)	278 (55)	50 (59)	69 (49)	89 (54)	0.44
CAD, *n* (%)	323 (64)	54 (64)	78 (55)	95 (58)	0.17
COPD, *n* (%)	54 (11)	13 (15)	23 (16)	13 (8)	0.08
Clinical presentation					
SBP (mmHg)	130 (114–144)	125 (114–148)	123 (105–141)	129 (109–145)	0.053
NYHA class, *n* (%)					< 0.001
I	110 (22)	15 (18)	23 (16)	10 (6)	
II	253 (50)	48 (56)	62 (44)	67 (41)	
III	136 (27)	22 (26)	56 (39)	86 (53)	
IV	3 (1)	0 (0)	1 (1)	0 (0)	
Peripheral oedema, *n* (%)					< 0.001
None	368 (73)	57 (67)	79 (56)	65 (40)	
Ankles	64 (13)	15 (18)	32 (22)	36 (22)	
> Ankles	72 (14)	13 (15)	31 (22)	63 (38)	
Crackles, *n* (%)					0.003
None	460 (91)	76 (90)	117 (82)	127 (77)	
Basal	39 (8)	8 (9)	23 (16)	33 (20)	
> Basal	5 (1)	1 (1)	2 (2)	4 (3)	
JVP, *n* (%)					< 0.001
Not visible	488 (97)	74 (87)	124 (87)	112 (68)	
Raised 1–4 cm	14 (3)	8 (9)	12 (9)	34 (21)	
Raised > 1–4 cm	3 (< 1)	3 (3)	6 (4)	18 (11)	
Hepatomegaly, *n* (%)					< 0.001
Not palpable	494 (98)	79 (93)	134 (94)	144 (88)	
Palpable	10 (2)	6 (7)	8 (6)	20 (12)	
Blood results					
Haemoglobi*n* (g/dL)	13.4 (12.3–14.6)	13.1 (12.0–14.1)	13.4 (12.3–14.4)	12.6 (11.3–13.6)	< 0.001
Albumin (g/L)	39 (37–40)	38 (36–40)	38 (36–40)	37 (35–40)	< 0.001
Urea (mmol/L)	6.8 (5.2–9.4)	7.0 (5.2–10.4)	7.4 (5.8–9.8)	8.9 (6.3–12.2)	< 0.001
eGFR (mL/min/1.73 m^2^)	64 (48–80)	63 (49–79)	57 (43–79)	53 (41–76)	0.001
Bilirubin (mmol/L)	13 (11–17)	15 (12–20)	16 (13–20)	17 (13–22)	< 0.001
ALT (U/L)	21 (16–27)	20 (17–28)	20 (16–26)	19 (15–25)	0.12
ALP (U/L)	72 (59–86)	71 (58–86)	78 (63–95)	81 (65–107)	< 0.001
hsCRP (mg/L)	3.1 (1.3–6.1)	3.0 (1.6–8.4)	3.5 (1.6–9.0)	5.2 (1.7–9.3)	0.006
NT-proBNP (pg/mL) (overall population)	571 (262–1264)	1573 (803–2714)	2072 (958–3935)	3313 (1750–5900)	< 0.001
NT-proBNP (pg/mL) (patients without AF)	464 (220–1060)	1437 (567–2884)	2030 (680–3693)	4173 (1526–8579)	< 0.001
NT-proBNP (pg/mL) (patients with AF)	1122 (575–1865)	1746 (1178–2691)	2152 (1152–4515)	3146 (1771–4735)	< 0.001
Treatment at referral					
BB, *n* (%)	394 (78)	68 (80)	102 (72)	117 (71)	0.15
ACE-I/ARBs, *n* (%)	437 (87)	64 (75)	117 (82)	132 (81)	0.027
MRAs, *n* (%)	162 (32)	27 (32)	52 (37)	54 (33)	0.79
PMK, *n* (%)	52 (10)	12 (14)	24 (17)	28 (17)	0.06
ICD, *n* (%)	38 (8)	9 (11)	17 (12)	13 (8)	0.35
CRT, *n* (%)	21 (4)	7 (8)	13 (9)	11 (7)	0.09
Loop diuretic—furosemide equivalent dose					
No loop diuretic, *n* (%)	180 (36)	27 (32)	34 (24)	33 (20)	< 0.001
1–40 mg/day	215 (43)	32 (38)	66 (46)	60 (37)	
41–80 mg/day	77 (15)	18 (21)	24 (17)	40 (24)	
> 80 mg/day	32 (6)	8 (9)	18 (13)	31 (19)	

*BMI* body mass index, *CAD* coronary artery disease, *PMK* pacemaker, *ICD* implantable cardioverter defibrillator, *CRT* cardiac resynchronisation therapy, *COPD* chronic obstructive pulmonary disease, *SBP* systolic blood pressure, *NYHA* New York heart association, *JVP* jugular venous pressure, *eGFR* estimated glomerular filtration rate, *ALT* alanine aminotransferase, *ALP* alkaline phosphatase, *HsCRP* High sensitivity C-reactive protein, *NT-proBNP* amino terminal pro-brain natriuretic peptide, *BB* beta blockers, *ACE-I/ARBs* angiotensin converting enzyme inhibitors/angiotensin II receptor blockers, *MRAs* mineralocorticoid receptor antagonists

### Electro- and echo- cardiographic characteristics (Table 2)

**Table 2:** Baseline ECG and echocardiographic characteristics of patients with heart failure (HF) according to tricuspid regurgitation velocity (TRV) and inferior vena cava (IVC) diameter

Variable	TRV ≤ 2.8 m/s IVC < 21 mm (*n* = 504)	TRV > 2.8 m/s IVC < 21 mm (*n* = 85)	TRV ≤ 2.8 m/s IVC ≥ 21 mm (*n* = 142)	TRV ≥ 2.8 m/s IVC ≥ 21 mm (*n* = 164)	*P*-value
ECG					
HR (bpm)	70 (61–79)	68 (60–80)	72 (62–80)	70 (60–80)	0.26
QRS interval (ms)	104 (92–134)	104 (88–134)	110 (94–142)	119 (97–154)	0.31
QTc interval (ms)	440 (412–460)	430 (410–470)	440 (420–470)	450 (420–480)	0.001
AF, *n* (%)	105 (21)	30 (35)	78 (55)	102 (62)	< 0.001
Echocardiography					
LVEDD (mm)	56 (49–62)	52 (48–61)	58 (50–64)	59 (51–66)	0.002
LVEDV (mL)	137 (101–180)	121 (92–178)	150 (100–217)	162 (113–212)	0.001
LVEF (%)	44 (36–53)	50 (32–60)	42 (31–55)	41 (32–56)	0.15
LVEF ≥ 50%, *n (%)*	169 (34)	43 (51)	54 (38)	62 (38)	< 0.001
GLS (%)	− 10.4 (− 13.6, − 7.6)	− 11.5 (− 15.3, − 6.4)	− 9.0 (− 12.4, − 5.7)	− 8.4 (− 12.9, − 5.5)	0.002
LA diameter (mm)	41 (38–46)	43 (39–47)	45 (41–50)	48 (43–52)	< 0.001
LA area (cm^2^)	21 (18–26)	24 (20–30)	28 (24–31)	30 (26–34)	< 0.001
LA volume (mL)	65 (49–87)	79 (62–105)	90 (68–114)	106 (87–131)	< 0.001
LAVi (mL/m^2^)	33 (25–45)	47 (34–63)	47 (35–60)	59 (47–72)	< 0.001
TAPSE (mm)	20 (16–22)	18 (14–21)	17 (14–21)	15 (13–17)	< 0.001
IVC (mm)	16 (15–18)	18 (17–19)	23 (22–26)	24 (23–27)	NA
Mitral regurgitation, *n* (%)					< 0.001
None/trivial	334 (66)	31 (36)	62 (44)	37 (23)	
Mild	131 (26)	37 (44)	53 (37)	72 (44)	
Moderate/severe	39 (8)	17 (20)	27 (19)	54 (33)	
Tricuspid regurgitation, *n* (%)					< 0.001
None/trivial	433 (86)	36 (42)	86 (60)	38 (23)	
Mild	66 (13)	41 (48)	37 (26)	79 (48)	
Moderate/severe	5 (1)	8 (10)	19 (14)	47 (29)	
TRV (m/s)	2.24 (2.12–2.50)	3.04 (2.89–3.28)	2.50 (2.34–2.69)	3.16 (3.00–3.49)	NA
TRV—not measurable, *n (%)*	16 (3)	0 (0)	5 (4)	0 (0)	NA

*HR* heart rate, *AF* atrial fibrillation, *LVEDD* left ventricular end diastolic diameter, *LVEDV* left ventricular end diastolic volume, *LVEF* left ventricular ejection fraction, *GLS* global longitudinal strain, *LA* left atrial; *LAVi* left atrial volume index, *TAPSE* tricuspid annular plane systolic excursion

Heart rate and QRS duration on ECG were similar amongst groups, but those with both high TRV and dilated IVC had a longer QTc interval and were more likely to have atrial fibrillation. LVEF was similar amongst groups; however, patients with high TRV but normal IVC were more likely to have an LVEF ≥ 50%. Patients with both high TRV and dilated IVC had greater impairment of longitudinal systolic function, as measured by GLS (less negative), greater left ventricular and atrial size, lower tricuspid annular plane systolic excursion (TAPSE) and were more likely to have moderate/severe mitral or tricuspid regurgitation than others.

### Outcomes

During a median follow-up of 860 days (435–1121), 239 patients with heart failure died and 346 died or were hospitalised with worsening heart failure.

**Fig. 1:**
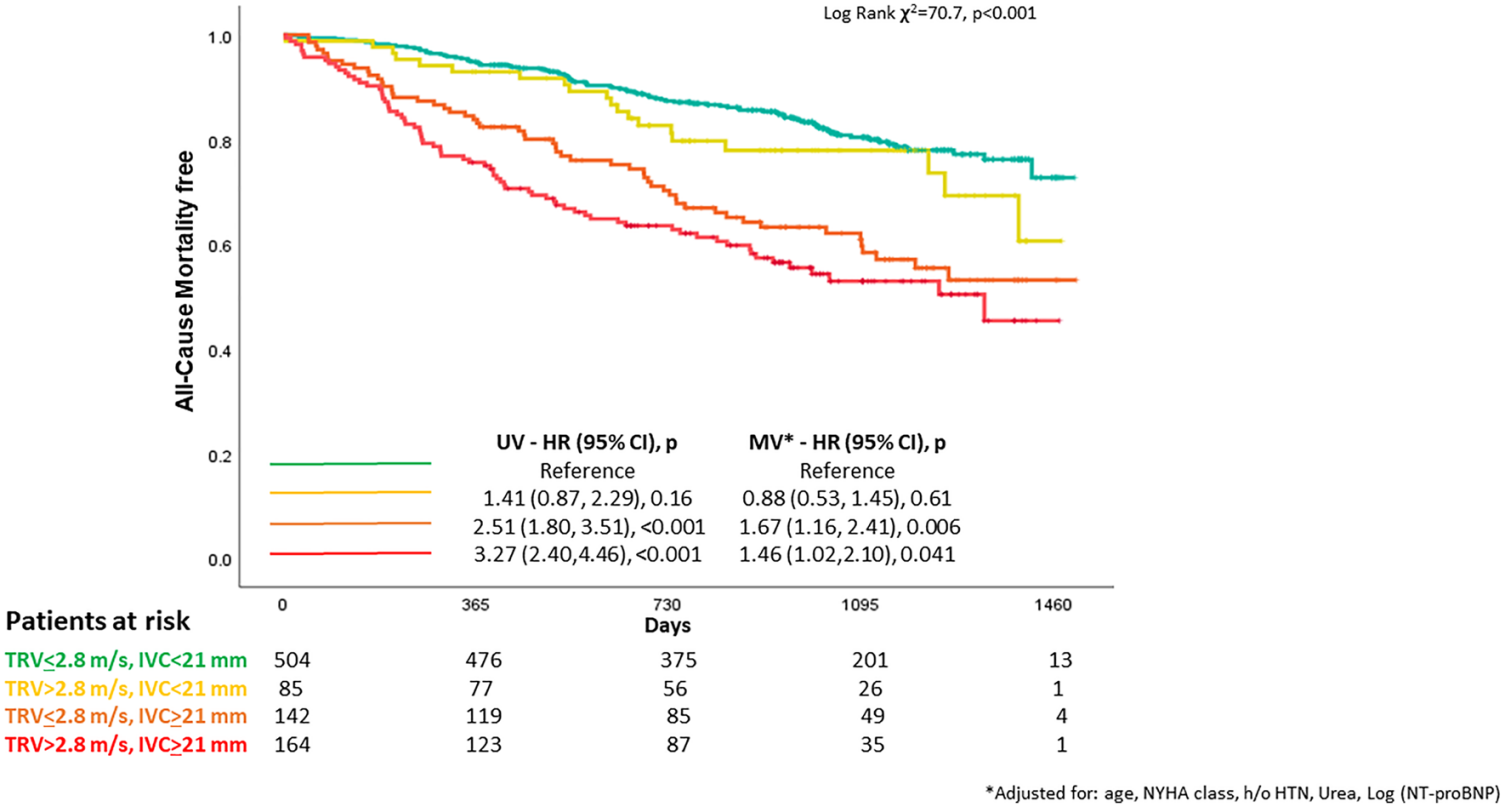
Compared to patients with both normal IVC and TRV (reference, in green), those with high TRV but normal IVC (in yellow) had a similar mortality, whilst those with a dilated IVC but normal TRV (in orange) had a higher risk. Patients with both a dilated IVC and high TRV had the greatest risk (in red)

**Fig. 2:**
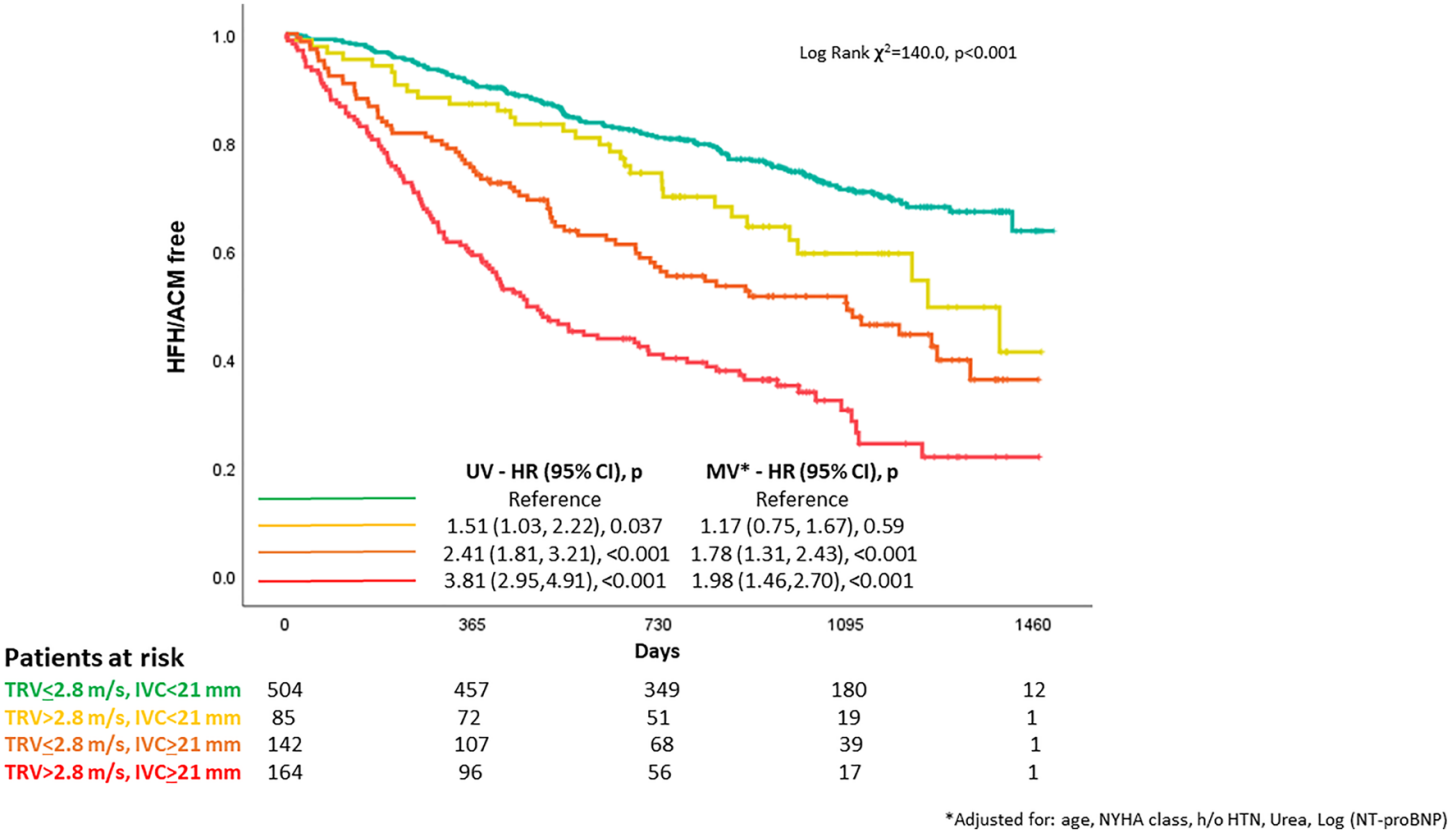
In univariable (UV) analysis, compared to patients with both normal IVC and TRV (reference, in green), those with high TRV but normal IVC (in yellow) had a greater risk of death or heart failure hospitalisation. When the model was adjusted (MV) for age, NYHA class, history of hypertension (HTN), urea and NT-proBNP, only those with a dilated IVC (in orange and red) had a greater risk of poor prognosis, regardless of TRV. Patients with both a dilated IVC and high TRV had the greatest risk (in red)

Compared to patients with both normal TRV and IVC, those with high TRV but normal IVC had similar risk of death (Fig. 1) and, in univariable analysis only, more than a 50% greater risk of a combined event (Fig. 2). Compared to others, those with a dilated IVC had a greater risk of both outcomes, regardless of TRV, even after multivariable adjustment. Those with both a high TRV and a dilated IVC had the greatest risk, with more than half being admitted with heart failure or dying within two years from the baseline clinical visit (*graphical abstract*). Findings were similar amongst patients with a reduced (≤ 40%, HFrEF) or higher (> 40%) LVEF (supplementary Fig. 1 and 2).

## Discussion

Our study suggests that, among ambulatory patients with heart failure, IVC diameter can be measured by expert echocardiographers in the vast majority. Importantly, in the present paper, we expand on previous analyses and have found that patients with a dilated IVC are at greater risk of heart failure hospitalisation or death even when TRV is normal.

Up to half of ambulatory patients with heart failure have a raised PAP, which is associated with an adverse prognosis [16]. Treatment strategies targeting high PAP in patients with HF have been widely tested with mixed results. Sodium-glucose cotransporter 2 inhibitors or the angiotensin receptor II blocker—neprilysin inhibitor might reduce PAP in patients with heart failure [16, 17, 18], although there is some disparity amongst trials [19]. Endothelin receptor antagonists might be of benefit for patients with primary PHT, but for patients with HF they cause water and salt retention leading to weight gain and oedema and have not been shown to improve outcomes [20]. Phosphodiesterase inhibitors also failed to improve symptoms or exercise capacity [21]. Inter-atrial septal devices, designed to decompress the left atrium during exercise by shunting blood into the right atrium, may have adverse effects on symptoms and prognosis if PHT is fixed, although patients whose pulmonary vascular resistance drops during exercise might benefit [22].

Control of congestion is the most effective intervention for reducing PAP and controlling symptoms and signs of HF and diuretics are the most important means of controlling congestion [23, 24]. Invasive pressure monitoring shows that an increase in PAP precedes changes in body weight or symptoms of worsening congestion by several weeks; increasing diuretic doses in response to increases in PAP reduces the risk of HF hospitalisations, although this has not yet been shown to translate into a reduction in death [16, 23, 24]. The failure to demonstrate a substantial effect of PAP guided therapy on outcomes may be because intervention only reduced mean PAP by about 1 mmHg. Whether more robust intervention would be more effective or whether it would be limited by side-effects such as systemic arterial hypotension and renal dysfunction is uncertain but needs to be tested. In the meantime, invasive monitoring technologies are not widely available due to their high costs [16].

The European Society of Cardiology (ESC) and the European Respiratory Society (ERS) have recently recommended lowering the cut-off for the diagnosis of PHT from a mean pulmonary arterial pressure (mPAP) of 25 mmHg to 20 mmHg at rest on haemodynamic assessment by right heart catheterisation [10] but, in concert with ESC heart failure guidelines, [13] they still state that if the TRV is either not measurable or ≤ 2.8 m/s on echocardiography the probability of pulmonary hypertension or raised LV filling pressures is low. However, these recommendations do not consider the possibility that PAP may not be elevated despite a high pulmonary vascular resistance when cardiac output is low. Also, when the right ventricular myocardium is diseased, its ability to sustain even a modest increase in PAP may be compromised, resulting in further dilation of the right ventricle and tricuspid ring. This results in worsening tricuspid regurgitation; paradoxically, measures of right ventricular dysfunction, such as TAPSE, may improve as the right ventricle offloads its contents into the venous circulation. [25] Furthermore, PAP pressure often rises during exercise in the presence of pulmonary vascular dysfunction. [22]

Data from the VA-CART programme (21,727 patients who underwent right heart catheterisation, mostly men, of whom ~ 55% had a history of heart failure) [26] suggest that the risk of hospitalisation and death starts to increase, when mPAP rises above 18 mmHg. A meta-analysis of 15 studies with more than 16,000 patients who underwent right heart catheterisation or echocardiography (N = 11,749 and 4,733, respectively) suggested that the risk of death increases for measured or calculated mPAP of 19–24 mmHg [27]. It is difficult to relate these measures to findings obtained at echocardiography. Although frequently used to estimate PAP, TRV allows only an estimate to be made of the systolic pressure drop from the right ventricle to the right atrium; adding an estimate of RAP to approximate sPAP by ultrasound is prone to error as it depends on assumptions of the relation between IVC size and RA pressure. Estimating mean pulmonary artery pressure from the TRV is even more likely to be fraught with error. In contrast, measuring IVC diameter is simple in any setting (including with hand-held devices) and, amongst expert sonographers, intra- or inter-operator variability is low [11, 28]. IVC diameter integrates information about intravascular volume, right ventricular function and right atrial pressure. Combining the assessment of TRV with IVC might make it possible to improve the specificity and sensitivity of a PHT diagnosis and to improve risk stratification.

Most of the blood in the circulation is contained in the venous system, which is highly compliant and can buffer the effects of large increases in intravascular volume. This might retard the rise in TRV secondary to volume expansion. In animal models, PAP only increases once the capacity of the venous system has been overwhelmed [12]. In a small mechanistic trial, an increase in IVC size, but not a rise in TRV measured by ultrasound, was associated with worsening congestion in ambulatory patients with mild HF after temporary suspension of medical therapy [29]. A rapid reduction in IVC diameter (by around 10–15% within 1 h, potentially lasting up to 2–3 h) follows intravenous administration of furosemide in patients admitted with acute heart failure [30], potentially reflecting veno-dilatation rather than increased natriuresis [31]. A series of observational studies conducted in patients admitted with acute heart failure reported that IVC diameter might track improvement in clinical congestion from admission to discharge, and that an engorged IVC diameter at discharge or at a routine visit predicts risk of an early readmission or death [32, 33]. Trials are ongoing to test the hypothesis that treatment guided by invasive or echocardiographic assessment of IVC size can improve the management of congestion in patients with HF [34, 35].

### Limitations

Our study has several limitations. These results represent the experience of a single, high-volume heart failure centre, and generalisability might be limited. We did not measure, invasively, pulmonary artery systolic pressure, nor did we assess changes in TRV during stress. Findings might be different for patients who are acutely unwell. We did not estimate right atrial pressure from echocardiographic variables: this is not so much a weakness as a deliberate feature of the present study.

## Conclusion

On routine echocardiography at an out-patient clinic, more than one third of ambulatory patients with HF have a dilated IVC, of whom about half have a normal TRV. A dilated IVC is associated with a worse prognosis even when TRV is normal. By contrast, a high TRV alone is not associated with a greater risk of heart failure hospitalisation or death. A dilated IVC might be a clinically useful therapeutic target in patients with heart failure, regardless of TRV.

## Supplementary Information

Below is the link to the electronic supplementary material.

Supplementary Figure 1 supplementary. Compared to patients with both normal IVC and TRV (reference, in green), those with a dilated IVC (in orange and red) had a higher risk of mortality, regardless of TRV or left ventricular ejection fraction (LVEF). Those with high TRV but normal IVC (in yellow) had a greater risk only amongst those whose LVEF was <40%. file1 (TIF 161 KB)

Supplementary Figure 2 supplementary. In univariable (UV) analysis, compared to patients with both normal IVC and TRV (reference, in green), those with high TRV but normal IVC (in yellow) had a greater risk of death or heart failure hospitalisation only amongst those whose left ventricular ejection fraction (LVEF) was >40%. When the model was adjusted (MV) for age, NYHA class, history of hypertension (HTN), urea and NT-proBNP, only those with a dilated IVC (in orange and red) had a greater risk or poor prognosis, regardless of TRV or LVEF. Patients with both a dilated IVC and high TRV had the greatest risk (in red). file2 (TIF 166 KB)

## Data Availability

The dataset analysed for the current study is available from the corresponding author on reasonable request. Applicants will be required to obtain all necessary permissions relevant to data-protection regulations before access to data is granted.
