# Supplementary figures and images for: Inferior vena cava diameter is associated with prognosis in patients with chronic heart failure independent of tricuspid regurgitation velocity
Source: Clin Res Cardiol. 2023 Mar 10;112(8):1077–86. doi: 10.1007/s00392-023-02178-4 (PMC10359207; doi:10.1007/s00392-023-02178-4)

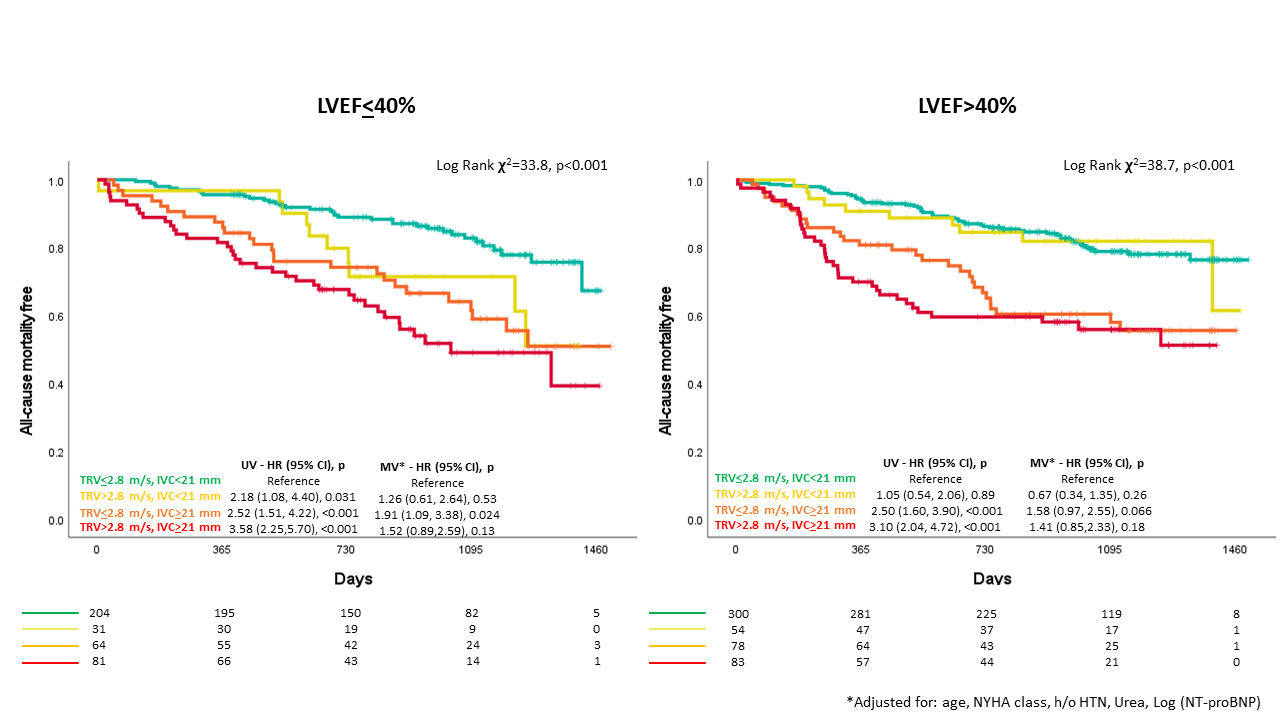

Supplement: Supplementary file 1 — Supplementary Figure 1 supplementary. Compared to patients with both normal IVC and TRV (reference, in green), those with a dilated IVC (in orange and red) had a higher risk of mortality, regardless of TRV or left ventricular ejection fraction (LVEF). Those with high TRV but normal IVC (in yellow) had a greater risk only amongst those whose LVEF was <40%. file1 (TIF 161 KB) [file 392_2023_2178_MOESM1_ESM.tif]

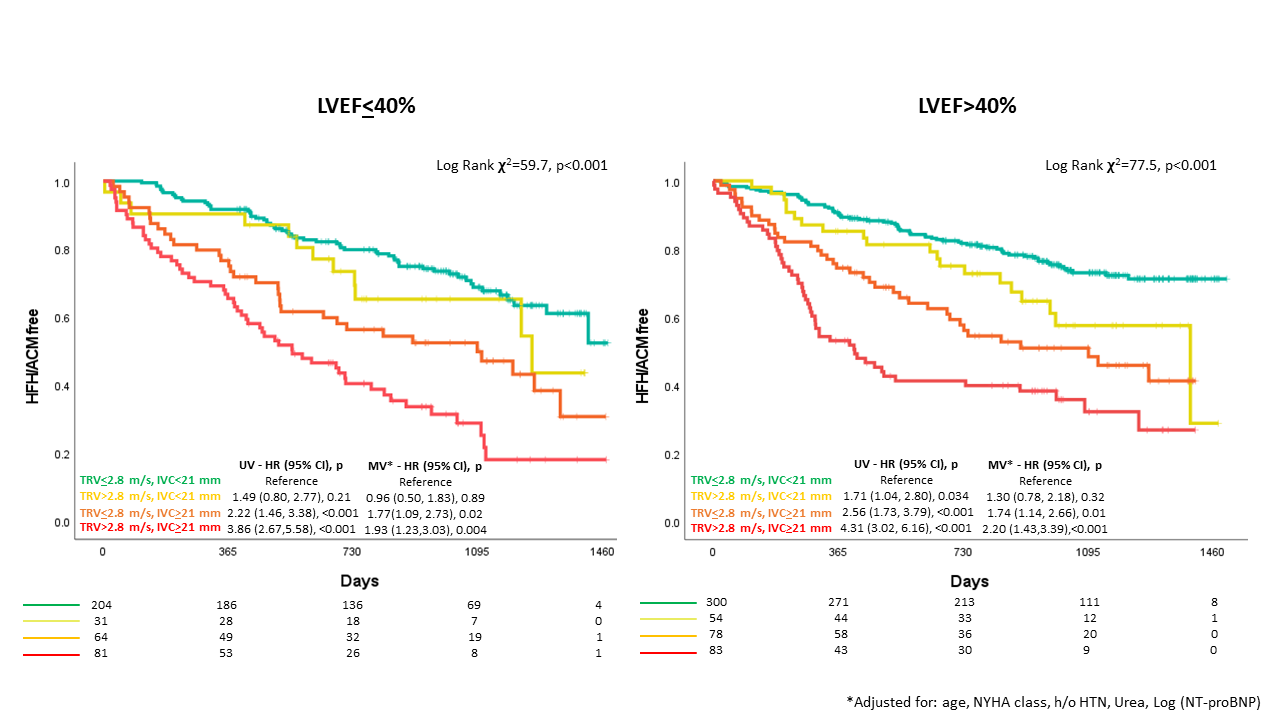

Supplement: Supplementary file 2 — Supplementary Figure 2 supplementary. In univariable (UV) analysis, compared to patients with both normal IVC and TRV (reference, in green), those with high TRV but normal IVC (in yellow) had a greater risk of death or heart failure hospitalisation only amongst those whose left ventricular ejection fraction (LVEF) was >40%. When the model was adjusted (MV) for age, NYHA class, history of hypertension (HTN), urea and NT-proBNP, only those with a dilated IVC (in orange and red) had a greater risk or poor prognosis, regardless of TRV or LVEF. Patients with both a dilated IVC and high TRV had the greatest risk (in red). file2 (TIF 166 KB) [file 392_2023_2178_MOESM2_ESM.tif]
